# Genome-wide association susceptibility loci for cluster headache support a role for inflammation in the pathophysiology

**DOI:** 10.1186/s10194-026-02485-x

**Published:** 2026-08-04

**Authors:** Caroline Ran, Clémence Deborgies Sanches, Julia Swedblom, Katrin Wellfelt, Alessandro Antoniazzi, Joseph Lloyd, Felicia Jennysdotter Olofsgård, Stefan Spulber, Elisabet Waldenlind, Maria Lantz, Anna Steinberg, Anna Sundholm, Christina Sjöstrand, Andrea Carmine Belin

**Affiliations:** 1 Centre for Cluster Headache, Department of Neuroscience, Biomedicum D7, Karolinska Institutet, Stockholm, 171 77 Sweden; 2 Department of Neurology, Karolinska University Hospital, Stockholm, 171 76 Sweden; 3 Deparment of Clinical Neuroscience, Karolinska Institutet, Stockholm, 171 77 Sweden; 4 Department of Clinical Sciences, Danderyd Hospital, Karolinska Institutet, Stockholm, 182 88 Sweden

**Keywords:** GWAS, Genetic, SNP, Inflammasome, Inflammation, Survival, Apoptosis

## Abstract

**Background:**

Recent genetic findings have broadened the perspectives of cluster headache pathophysiology. Genes identified by genome wide association studies are likely to be involved in the pathophysiological mechanisms underlying the disease. In this study, we performed validation and characterization of eight loci corresponding to nine genes identified in previous genome wide association studies.

**Methods:**

Genetic loci were validated by means of a case-control study using TaqMan genotyping in 671 individuals, and a meta-analysis using previously genotyped data. Pyrosequencing methylation analysis and reverse transcription quantitative PCR gene expression studies was performed in a subset of individuals with cluster headache and healthy controls.

**Results:**

Genetic associations were found with functional variants in two genes; *FHL5* and *MERTK.* The minor allele of the *FHL5* variant rs2273621 was associated with an increased risk of cluster headache in the genotyped samples (odds ratio = 1.42, *P* = 0.03) and in a meta-analysis (odds ratio = 1.29, confidence interval: 1.09–1.52), and the minor allele of the rs2230515 variant in *MERTK* was associated with lower risk in the meta-analysis (odds ratio = 0.69, confidence interval: 0.52–0.92). Methylation analysis further revealed altered methylation status of *MERTK*. Moreover, changes in relative gene expression were found for several candidate genes: a decrease in the mRNA expression of *DUSP10* (*P* < 0.001), *CAPN2* (*P* < 0.001), *UFL1* (*P* < 0.001) and *LRP1* (*P* = 0.007) in blood, and a decrease of *CAPN2* (*P* < 0.001) as well as an increase in the mRNA expression of *FTCDNL1/FONG* (*P* = 0.004) in fibroblasts in CH vs. controls. Only two genes; *PLCE1* and *WNT2,* could not be validated in the biological tissue studied; *WNT2*, possibly due to limited power, while *PLCE1* was not expressed in the available tissue.

**Conclusions:**

These results confirm the validity of seven candidate genes for cluster headache identified through GWAS. Furthermore, five of the candidate genes are abundantly expressed in both immune and blood cells, converge functionally on inflammatory signalling pathways and suggest a potential role for inflammasomes in cluster headache.

**Supplementary Information:**

The online version contains supplementary material available at 10.1186/s10194-026-02485-x.

## Introduction

Cluster headache (CH) is the third most common primary headache disorder following tension type headache and migraine. Heredity has been described for CH, but less frequently than for example for migraine [1]. It is not uncommon for individuals with CH to report that close relatives also have received a CH diagnosis, especially for females with CH (15% vs. 7% in males) [2]. Co-occurrence of CH has been reported in monozygotic twins, and first- and second-degree relatives of individuals with CH have increased risk of developing CH (5–39 times and 8 times increased risk respectively) [3].

To understand the hereditary component of CH etiology, numerous studies have investigated specific candidate genes (for review see [4, 5]) alongside genome wide association studies (GWAS) [6–8]. Furthermore, a meta-GWAS on 4,777 cases from ten European cohorts and one East Asian cohort was published in 2023 [9]. Seven risk loci were identified in the European CH cohorts encompassing or close to the following genes; dual specificity phosphatase 10 (*DUSP10*) on chromosome 1, MER proto-oncogene tyrosine kinase *(MERTK)* and formiminotransferase cyclodeaminase N-terminal like (*FTCDNL1* or *FONG*) on chromosome 2, four and a half LIM domains 5 (*FHL5*) alternatively UFM1 Specific Ligase 1 (*UFL1*) both mapped to the same loci on chromosome 6, wingless-type MMTV integration site family, member 2 (*WNT2*) on chromosome 7, phospholipase C epsilon 1 (*PLCE1*) on chromosome 10, and low-density lipoprotein receptor-related protein 1 (*LRP1*) on chromosome 12. In the East Asian cohort one additional risk loci was identified: Calpain-2 catalytic subunit (*CAPN2*) on chromosome 2 [9].

The most significant loci in all three GWASs as well as in the meta-GWAS was *MERTK*, encoding a receptor protein of the TYRO3/AXL/MER (TAM) receptor kinase family. MERTK has been described to play a role in many physiological processes such as cellular survival, proliferation, inflammation, tissue repair and efferocytosis, but the role of this gene in CH pathophysiology is still unknown. In a previous study, a first validation was made of *MERTK* as a CH candidate gene by gene and protein expression analysis in human and rat tissue. *MERTK* mRNA levels were found to be increased in individuals with CH as compared to controls. Additionally, Galectin-3 (Gal-3), a MERTK ligand, was elevated in CH. Presence of MERTK and Gal-3 were further verified in trigeminal ganglion (TG) from rats [10].

The loci identified through GWAS harbour genes with variable functions. It is important to validate and characterize these genes in terms of headache pathophysiology to identify the true disease-causing genes, and to better understand the biological implications of these genes and related pathways. Three of the GWAS loci, *FHL5*, *PLCE1* and *LRP1* overlap with known migraine GWAS loci, but the exact role in headache pathophysiology is still unclear [9]. *FHL5* is expressed in brain endothelial cells and has, similar to *MERTK*, been shown to activate the cAMP response element-binding protein (CREB) pathway, resulting in the formation of phosphorylated-CREB (P-CREB) [11]. P-CREB, has been identified in rat trigeminal nucleus caudalis and reported to increase the excitability of nociceptive neurons in the trigeminovascular system [12]. *PLCE1* is part of the PLC enzyme family which regulates cellular communication. *PLCE1* triggers a cellular cascade by hydrolysing cell surface phospholipid phosfatidylinositol-4,5-bisfosfate (PIP2), resulting in elevated intracellular concentration of Ca^2+^ released from endoplasmic and sarcoplasmic reticulum [13]. It has been suggested that *PLCE1* is involved in neuroinflammation by prolonging signalling to secondary messengers, through the activation of the nuclear factor kappa B (NF-κB) pathway [14]. *LRP1* is a cell surface receptor which plays a complex role in the immune system and is involved in the maintenance of vascular integrity and neuronal function. Similarly to *MERTK*, it plays a crucial role in efferocytosis [15]. The remaining four loci have no known established link to headache disorders. *DUSP10*, an anti-inflammatory gene, plays an essential role in both innate and adaptive inflammatory response [16]. *FTCDNL1* is a gene encoding a formiminotransferase predicted to be involved in folate binding. Genetic variants located in *FTCDNL1* have been linked to osteoporosis and schizophrenia [17, 18]. *CAPN2* encodes a subunit of a non-lysosomal cysteine protease enzyme, Calpain-2. Calpains are Ca^2+^ dependent proteases which are involved in a multitude of pathways such as cytoskeleton remodelling and apoptosis [19]. *WNT2*, encodes a signalling molecule binding to the frizzled receptor family, it is known to be involved in embryogenesis and oncogenesis [20].

In this report we aimed to validate the CH GWAS loci identified in a previous meta-GWAS [9], by investigating gene expression of the genes mapped to each locus in tissue from individuals with CH and controls (excluding *MERTK* which was validated in another study) [10], and further to investigate methylation and functional variants previously identified by post-GWAS analysis at the *MERTK*, *FHL5* and *PLCE1* loci [7, 9].

## Materials and methods

The study design is summarized in Supplementary Fig. 1.

### Materials

All study participants have been recruited through Karolinska University Hospital and are part of the biobank at the Centre for Cluster Headache, Karolinska Institutet. Written informed consent from all study participants was obtained before being enrolled in the study. The study has been approved by the Swedish Ethical Review authority (ref: 2014/656 − 31), and all experiments were performed according to the declaration of Helsinki.

### Genetic analysis

#### Genotyping

The GWAS cohort consists of 542 individuals with CH recruited to the cluster headache biobank at the Centre for Cluster Headache at Karolinska Institutet between 2014 and 2020 and 296 controls previously included in GWAS [7, 9], the replication cohort consists of 165 individuals with CH recruited to the biobank between 2021 and 2025 and 506 controls not included in previous studies. The individuals with CH from the GWAS were re-genotyped in this study, as the markers of interest were previously included by imputation. Of the 165 new individuals with CH, 62.42% of the CH diagnoses have been validated by a neurologist in accordance with the ICHD 3rd edition [21], the remaining have self-reported CH. Controls were anonymous healthy blood donors (*n* = 467) or recruited through the Centre for Cluster Headache at Karolinska Institutet (*n* = 39). All study participants were asked to give a blood sample for subsequent DNA preparation using standard protocols and to fill out a questionnaire with personal and clinical information. The demographics of all participants are shown in Table 1.

**Table 1:** Demographics of analysed cohorts

Demographics	GWAS		Replication		Pyrosequencing		Gene expression fibroblasts		Gene expression blood	
All (n)	CH (542)	C (296)	CH (165)	C (506)	CH (62)	C (26)	CH (11)	C (9)	CH (20)	C (22)
Confirmed CH, % (n)	100 (542)	NA	62.42 (103)	NA	100 (62)	NA	100 (11)	NA	100 (20)	NA
Female, % (n)	31.6 (171)	39.53 (117)	42.42 (70)	48.4 (245)	53.8 (14)	51.6 (32)	18.2 (2)	33.3 (3)	10.0 (2)	45.5 (10)
Average age. years ± SD (n)	52.3 ± 14.57 (542)	43.6 ± 8.7 (11)	50.5 ± 24.2 (129)	41.5 ± 10.0 (28)	51 ± 13.6 (62)	42 ± 12.2 (26)	41.9 ± 15.7 (11)	47.8 ± 14.6 (9)	48.9 ± 15.3 (20)	38.6 ± 12.7 (22)
Average age of onset, years ± SD (n)	31.6 ± 17.3 (442)	NA	31.6 ± 18.3 (106)	NA	31.1 ± 12.4 (48)	NA	27.2 ± 15.1 (11)	NA	26.4 ± 8.5 (17)	NA
Chronic CH, % (n)	10.0 (54)	NA	17.0 (28)	NA	1.6 (1)	NA	27.3 (3)	NA	10.0 (2)	NA
Unknown subtype, % (n)	0.6 (3)	NA	26.7 (44)	NA	0	NA	0	NA	0	NA
Positive family history, % (n)	10.0 (54)	NA	11.5 (19)	NA	17.5 (10^a^)	NA	9.1 (1)	NA	27.8 (5^b^)	NA

CH = Cluster headache, C = Control, SD = Standard deviation, NA = not applicable/available. Confirmed CH = has CH diagnosis confirmed by neurologist, both confirmed (62.4%) and not confirmed (37.6%) CH cases were included in the replication cohort. Positive family history is defined as having a known relative with CH, Genetic controls were obtained through anonymous blood donations. ^a^ = information known for 57 individuals, ^b^ = information known for 18 individuals

The rs2230515 variant was selected for genotyping since it is in high linkage disequilibrium (LD) with the lead variant in *MERTK* identified previously [7]. Variants rs2273621 and rs2274224, both missense, were genotyped as they were reported to be in high LD with the intronic lead variants in *FHL5* and *PLCE1* in the previous meta-GWAS [9]. The Combined Annotation Dependent Depletion (CADD) scores for these genetic variants were: rs2230515 A > G; 0.166, rs2273621 A > G; 23.6 and rs2274224 G > C; 16.04 [7, 9, 22]. Genotyping was performed by quantitative PCR (qPCR) on a QuantStudio5™ (Applied Biosystems) instrument, using the recommended qPCR conditions, with 40 PCR cycles for all genetic variants. Approximately 10ng DNA was used, TaqMan^®^ Genotyping Assays (0.5X) (for Assay ID see Supplementary Table 1, Applied Biosystems), and TaqMan^®^ Genotyping Master Mix (1X) (Applied Biosystems) for each reaction, in a total volume of 10µL.

### Analysis of DNA methylation by pyrosequencing

DNA from whole blood of 62 individuals with CH and 26 controls (Table 1) was bisulfite converted using the EpiTect^®^ Bisulfite Kit (QIAGEN) according to the manufacturer’s instructions. The Pyrosequencing experiment was set up, and primers were designed using the PyroMark Assay Design software and the FASTA gene sequence where cysteine bases were replaced by thymine. Primers were purchased from Thermo Fisher, the reverse primer was biotinylated at the 5’-end (primer sequences are available in Supplementary Table 1). PCR was run on a BioRad thermocycler, with Kapa2G Fast DNA Polymerase (Merck), 0.2µL forward and reverse primer (10µM) and 1µL bisulfite converted DNA in a total reaction volume of 10µL. A touch-down program was used, with an initial step of activation (95 °C for 5 min) consecutive cycles of 95 °C for 20 s, 67 °C for 30 s reduced by 0.5 °C per cycle until 55 °C, and 72 °C for 30 s, followed by 25 cycles of 95 °C for 20 s, 55 °C for 30 s, 72 °C for 30 s, and last a termination step of 72 °C for 7 min. PCR product was verified on an agarose gel (3%) run at 75 V for 40 min.

The Pyro-methylation assay was verified by usage of an EpiTect Control DNA Set mixed to yield a methylated to unmethylated DNA ratio of 0, 25, 50, 75 and 100%. All three positions within the assay resulted in a linear relation to methylation status and could be included in further analysis (Supplementary Fig. 2).

PCR product was captured and denatured in 0.2 M NaOH using a vacuum-prep station. Single stranded DNA was washed and annealed with the sequencing primer at 80 °C for two min after which pyrosequencing was run on a PyroMark Q96 ID system using PyroMark GOLD Q96 reagents. Failed samples were re-run to obtain methylation data form all samples. Data was analysed using the software provided with the instrument.

### Gene expression

Fibroblast cell lines: Previously established primary fibroblast cell lines harvested 6 h after serum shock and frozen at -150 °C were used in this experiment. The cell lines were grown from skin biopsies obtained from 12 individuals with CH and 9 controls (Table 1). The full protocol of cell-line establishment and serum shock has been described in detail previously [23]. RNA was prepared from frozen cell pellets using the RNeasy Mini Kit (QIAGEN), in accordance with the manufacturer’s specifications. RNA concentrations were measured using NanoDrop 1000 (ThermoFisher).

Fresh blood cells: 10mL of venous blood was obtained in PAXgene blood RNA tubes (BD Sweden) from 20 individuals with CH and 22 control subjects (Table 1) and kept in -20 °C until further use. RNA was extracted from blood using the PAXgene^®^ Blood RNA Kit (PreAnalytiX,) according to the manufacturer’s instructions.

QuantiTect Reverse Transcription kit (QIAGEN) was used to prepare cDNA from total RNA. For each reaction 1000ng RNA from fibroblasts, and 500ng RNA from white blood cells was used as starting material. Gene expression was then analysed by Reverse Transcription quantitative PCR (RT-qPCR) using PowerUp™ SYBR^®^ Green Master Mix (ThermoFisher) and primers (500nM forward and reverse) for each target gene and two reference genes (TATA-Box Binding Protein (*TBP*) and Importin 8 (*IPO8*)). Primers were designed using primer 3 and NCBI primer design software [24–26], and purchased from ThermoFisher, primer sequences are available in Supplementary Table 1. RT-qPCR was run on a QuantStudio5™ (Applied Biosystems), in a total reaction volume of 10mL and using the settings recommended for the PowerUp™ master mix.

### Statistical analysis

Statistical analysis of genotype data was performed separately on both cohorts (GWAS and replication) and was then included in a meta-analysis using PLINK v1.9 [27]. Call rate was above 97% for all genetic variants (rs2230515 = 98.9%, rs2273621 = 97.4% and rs2274224 = 98.5%). Hardy-Weinberg equilibrium (HWE) analysis and an association analysis for the individuals included in the prior GWAS and the new samples were conducted separately. The association was analysed using a logistic regression with sex as a covariate. The analysis was corrected for multiple testing using Bonferroni correction with an α ≤ 0.05 considered significant. Last, a Meta-analysis was performed to compare results of the two groups. Forest plots were then generated in R version 4.5.1 (package: ‘forestplot’) using the PLINK output files of a basic case/control chi-square allele association test performed in the GWAS and replication cohort separately.

Methylation was analysed by comparing the % of methylation on each position analysed (1, 2 and 3). Data was tested for normality using Shapiro-Wilk test and analysed between groups with Wilcoxon’s test for non-normally distributed data, significant p-values were adjusted for three tests.

The analysis of gene expression was performed in QuantStudio™ Design and Analysis Software V2 (ThermoFisher). Gene expression was normalized to two reference genes (*IPO8* and *TBP*) and to a reference sample consisting of pooled cDNA from all controls. GraphPad QuickCalcs (GraphPad Software) was used to perform a Grubb’s test to detect outliers (https://www.graphpad.com/quickcalcs/). Two outliers were removed among individuals with CH; *FTCDNL1* in fibroblasts, and *UFL1* in blood, and one outlier among controls; *DUSP10* in blood. One control sample was removed from the *FTCDNL1* analysis in fibroblasts due to low CT confidence. The relative expression was Log transformed (log_2_) and normal distribution was evaluated using a Shapiro-Wilk test. Pairwise-differences were evaluated using Student’s t-test. False discovery rate (FDR) correction for multiple testing was performed using Benjamini-Hochberg correction at 0.05 level. All statistical analysis was performed in R version 4.5.1. Correlation analysis was performed using Pearson’s product-moment correlation in R by usage of the GGally package [28].

## Results

Three functional variants that had previously been highlighted as potential risk variants during post-GWAS analysis were screened in a Swedish cohort consisting of the GWAS cohort and a replication cohort. All three genetic variants, rs2230515, rs2273621 and rs2274224 were in HWE with *P* = 0.24, *P* > 0.99 and *P* = 0.33 respectively. There was no difference in the allele distribution of the *MERTK* variant rs2230515, and the *PLCE1* variant rs2274224 between CH and controls when analysed in the replication cohort (*P* = 0.32 and odds ratio (*OR*) = 0.77, with *95% c*onfidence interval (*CI*) 0.56–1.06, and *P* > 0.99 and *OR* = 0.93 with *95% CI* 0.71–1.23 respectively). The allele distribution of *FHL5* variant rs2273621 was significantly different between CH and controls in the replication cohort (*P* = 0.03 and *OR* = 1.42, with *95% CI* 1.08–1.87) (Table 2).

**Table 2:** Allele distribution of rs2273621, rs2274224 and rs2230515 in the replication cohort

Gene	Variant	Allele	Allele frequency % (n)		OR (95% CI)	Adj P-value
			Cluster headache	Controls		
MERTK	rs2230515	G	67.79 (221)	62.87 (635)	0.77 (0.56–1.06)	0.32
		A	32.21 (105)	37.13 (375)		
FHL5	rs2273621	A	59.26 (192)	66.36 (657)	**1.42 (1.08–1.87)**	**0.03**
		G	40.74 (132)	33.64 (333)		
PLCE1	rs2274224	G	59.81 (195)	59.34 (591)	0.93 (0.71–1.23)	> 0.99
		C	40.18 (131)	40.66 (405)		

Logistic regression (additive model) with sex as covariate was utilized to analyse allele association. OR = Odds ratio, CI = Confidence interval. Adj p-value = p-value adjusted for multiple testing using Bonferroni correction

A separate analysis of the allele association for these three markers in the GWAS cohort can be found in Supplementary Tables 2, and specific allele frequencies in validated respectively non validated CH cases are available in Supplementary Table 3. Briefly, there was no difference in the allele distribution for the variants rs2273621 and rs2274224, while the minor allele (A) of rs2230515 was found more often in controls than cases (*P* < 0.001 and *OR* = 0.6 with *95% CI* 0.48–0.76).

**Fig. 1:**
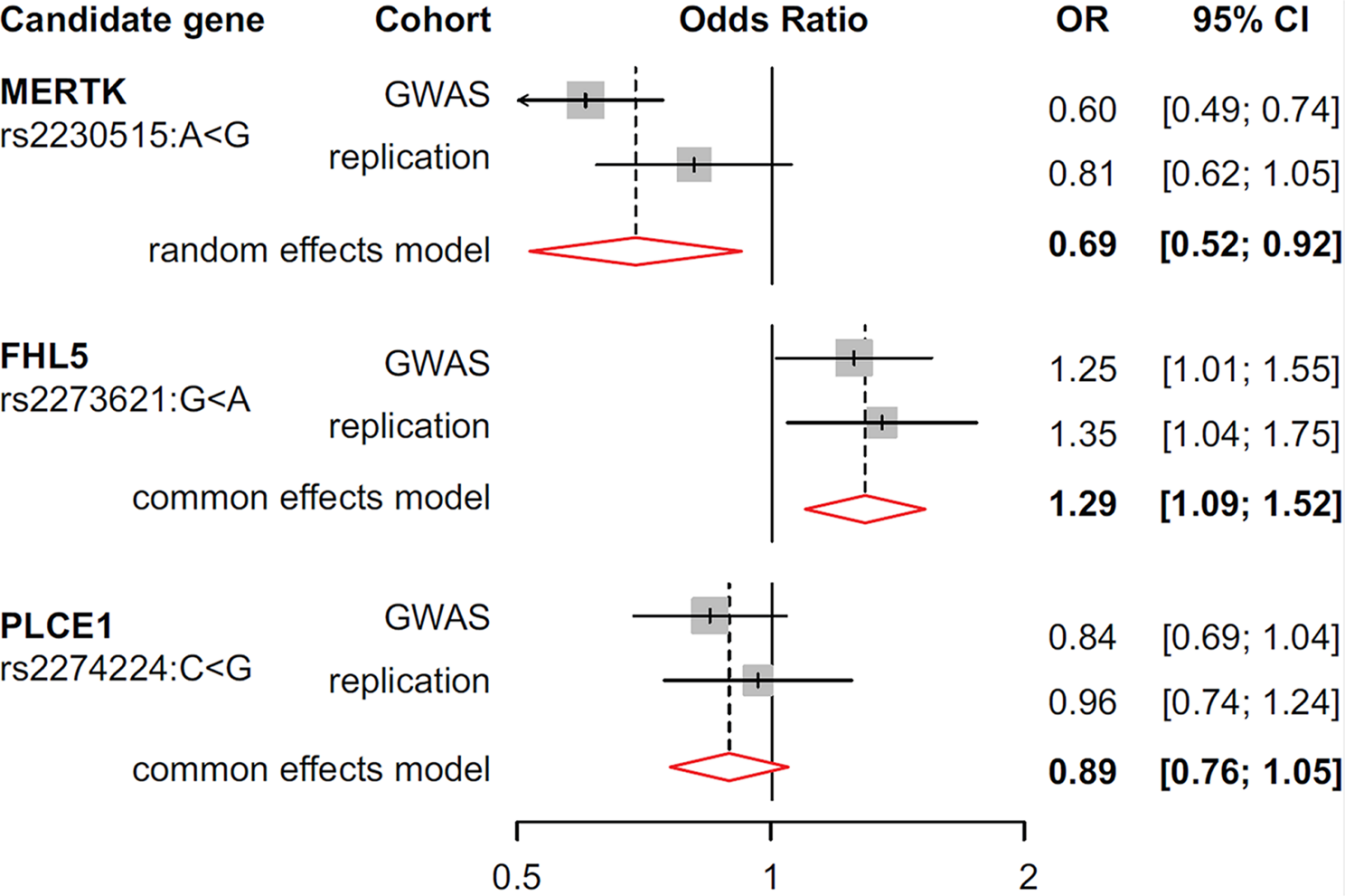
Meta-analysis, rs2230515, rs2273621, rs2274224. Forest plot of meta-analysis, random or common effects model, represented by the purple diamond, show the summarized effect of the studies. OR = Odds ratio from a chi-square test, 95% Cl = confidence interval, *MERTK* = MER proto-oncogene tyrosine kinase, *FHL5* = Four and a half LIM domains 5, *PLCE1* = Phospholipase C epsilon 1. *MERTK*: rs2230515:A < G, heterogeneity (I^2^) = 65.7%, 𝜏^2^= 0.029, *P* = 0.12. The weight of the GWAS cohort was 61.1% and the replication cohort was 38.9%. *FHL5*:rs2273621:G < A, heterogeneity (I^2^) = 0.0%, 𝜏^2^= 0, *P* = 0.002. The weight of the GWAS cohort was 59.8% and the replication cohort was 40.2%. *PLCE1*:rs2274224:C < G, heterogeneity (I^2^) = 0.0%, 𝜏^2^= 0, *P* = 0.15. The weight of the GWAS cohort was 60.3% and the replication cohort was 39.7%

Next, a meta-analysis was performed to analyse the occurrence of these three genetic variants of interest in a larger Swedish cohort. The *MERTK* variant rs2230515 showed substantial heterogeneity (*I*^*2*^) = 65.7%, indicating that the effect may vary between the two studies (GWAS and replication). Additionally, the tau squared (𝜏^2^) value was 0.029 for rs2230515, suggesting some variance of true effect. A random effects meta-analysis was run which revealed a significant protective effect of the rs2230515 A allele, with a summarized effect, *OR*, of 0.67 and a *95% CI* of 0.57–0.80 (*P* = 0.012) (Fig. 1). Furthermore, both rs2273621 and rs2274224 were found to have low heterogeneity (*I*^*2*^) (0.0%) and were run using a fixed effects model. The meta-analysis revealed a significant risk effect of the rs2273621 G allele (*OR* = 1.29, 1.09 < *OR* < 1.52, *P* = 0.002) (Fig. 1). No association was found for rs2274224 (*OR* = 0.89, 0.76 < *OR* < 1.05, *P* = 0.15) (Fig. 1). Both significant markers; rs2230515 in *MERTK* and rs2273621 in *FHL5* were confirmed as expression Quantitative Trait Loci (eQTL) in silico by searching the Genotype-Tissue Expression (GTEx) portal [29]. The protective A allele of rs2230515 (more common in controls) was reported to correlate with lower *MERTK* gene expression activity in arteries while the risk associated allele (G) of rs2273621 was correlated with lower *FHL5* gene expression in arteries (Supplementary Fig. 3).

**Fig. 2:**
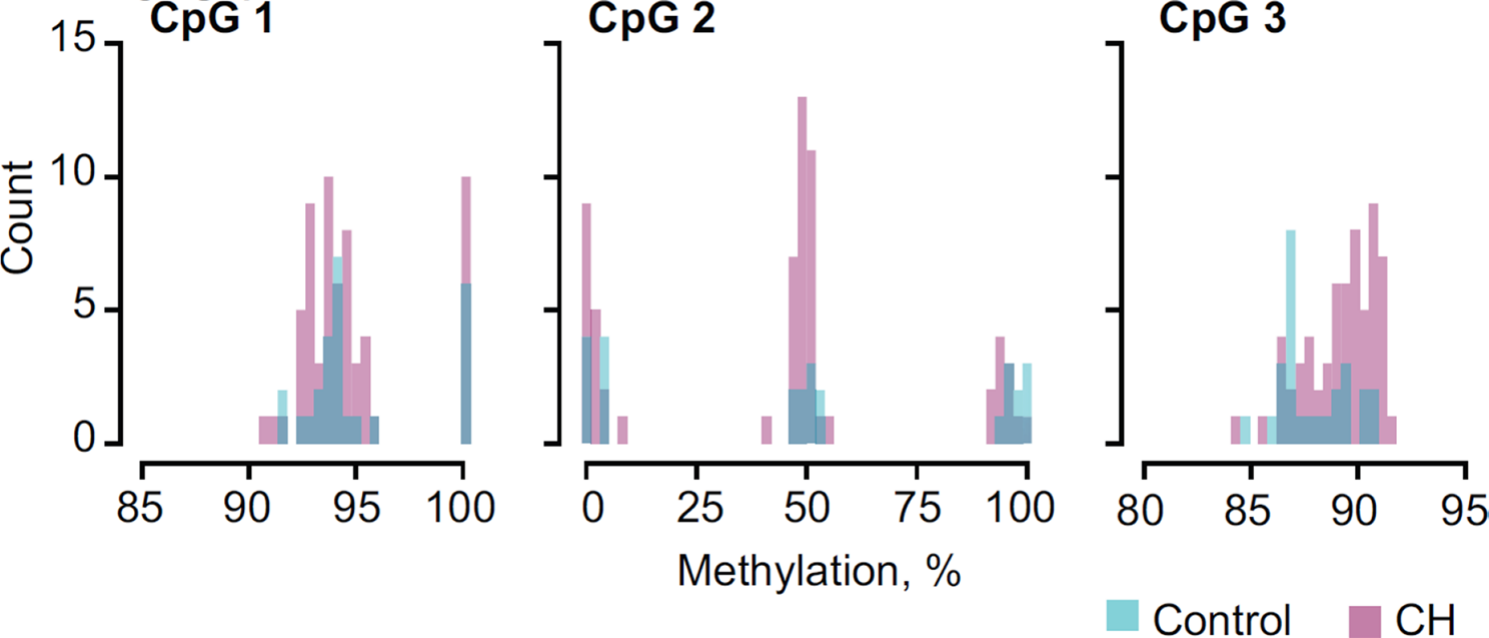
Analysis of three CpG sites in the *MERTK* gene. *MERTK* = MER proto-oncogene tyrosine kinase. Distribution of methylation on three CpG sites in the *MERTK* gene in individuals with cluster headache (CH), (*n* = 62) and controls (*n* = 26). On the y-axis; number of individuals, on the x-axis; percentage of methylation at each position; CpG 1, CpG 2 and CpG 3

In addition to the suggested functional variants analysed herein, a predicted methylation site within the *MERTK* gene was investigated, the cg05979619 CpG site at position 112,704,577 on chromosome 2 according to GRCh37 coordinates. Within the 219 bp fragment amplified by PCR there were three CpG positions to analyse for methylation (Supplementary Fig. 2). Position 2 corresponded to the predicted hypermethylated site cg05979619. Results showed that Position 1 and 3 were highly methylated (Fig. 2), while Position 2 had a wider distribution of methylation patterns (Fig. 2). Groupwise comparison showed that methylation was similar on Position 1 with an average methylation of 94.7% in CH as compared to 95.2% in controls (*P* = 0.56). At Position 2 the average methylation was lower in CH (44.5%) than in controls (51.7%), but the difference was not statistically significant (*P* = 0.22). At Position 3 there was a small but significant difference between CH and controls (89.2% vs. 87.9%, *P* < 0.001, adjusted *P*-value = 0.002).

**Fig. 3:**
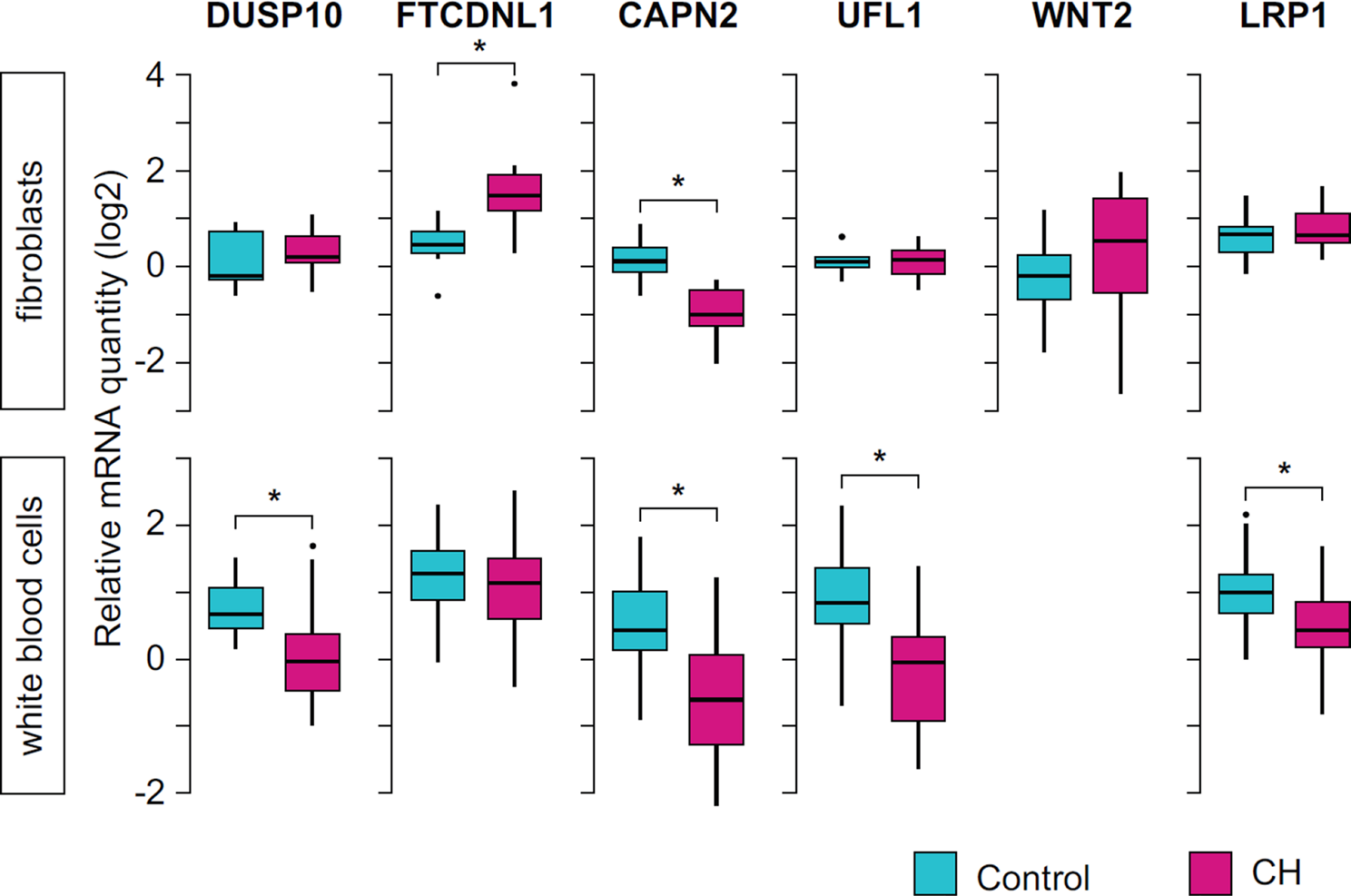
Candidate gene expression in fibroblast cell lines and in white blood cells. Top panel: relative gene expression in fibroblasts from 12 individuals with cluster headache (CH) and 9 controls. Bottom panel: relative gene expression in blood cells from 20 individuals with CH and 20 controls. Gene expression was normalized and log_2_ transformed, normal distribution was evaluated using a Shapiro-Wilk test and group differences evaluated with a Students’ t-test. *DUSP10*; dual specificity phosphatase 10, *FTCDNL1*; and formiminotransferase cyclodeaminase N-terminal like, *CAPN2*; Calpain-2 catalytic subunit, *UFL1*; UFM1 Specific Ligase 1, *WNT2*; wingless-type MMTV integration site family, member 2, *LRP1*; low-density lipoprotein receptor-related protein

Gene expression of *DUSP10*, *FTCDNL1*, *CAPN2*, *FHL5*, *WNT2*, *PLCE1* and *LRP1* were assessed in cDNA from human fibroblasts and white blood cells from individuals with CH and controls. *PLCE1* mRNA was not detected in either investigated tissue and *WNT2* was not detected in blood. *DUSP10*, *FTCDNL1*, *CAPN2*, *WNT2* and *LRP1* were all expressed in fibroblasts and were run in 9 control and 11 CH samples (Fig. 3). Furthermore, *DUSP10*, *FTCDNL1*, *CAPN2* and *LRP1* were expressed in blood cells and were analysed in 22 control and 20 CH samples (Fig. 3). All data are presented as relative gene expression after normalization of mRNA levels to reference genes and a reference sample and removal of outliers as indicated by Grubb’s test. Results are presented in Fig. 3.

The expression of *FTCDNL1* was increased in fibroblasts from individuals with CH (*P* = 0.004) and *CAPN2* relative mRNA levels was decreased (*P* < 0.001) as compared to controls (Fig. 3). Gene expression for *DUSP10*, *UFL1*, *WNT2* and *LRP1* was very similar between the CH and control groups in fibroblasts. As sample sizes were small, in particular for fibroblasts, small differences in gene expression between cases and controls may remain undetected by statistical tests. In blood cells, *FTCDNL1* did not differ in the CH group as compared to controls, while the remaining genes *DUSP10* (*P* < 0.001), *CAPN2* (*P* < 0.001), *UFL1* (*P* < 0.001) and *LRP1* (*P* = 0.007) were downregulated (Fig. 3). The largest difference between groups was found for *CAPN2*, with a consistent decrease of mRNA levels in CH in both blood and fibroblasts.

A correlation analysis was performed for each tissue (blood and fibroblasts) separately to investigate if the candidate genes vary in consistent patterns. In fibroblasts sparse correlation between the different mRNA transcripts was observed. Only one correlation was found in all the samples: a negative correlation between *FTCDNL1* and *CAPN2* (Supplementary Fig. 4). In blood cells there were extensive positive correlations between mRNA levels of almost all the candidate genes except for *FTCDNL1* (which was the only analysed gene that did not differ between individuals with CH and controls). *DUSP10* displayed positive correlations with *CAPN2*, *UFL1* and *LRP1.* Similarly, *CAPN2* displayed positive correlations with *DUSP10*, *UFL1* and *LRP1*. The two genes *UFL1* and *LRP1* did not correlate with each other but with the other candidate genes *DUSP10*,* FTCDNL1* and *CAPN2* (Supplementary Fig. 4).

## Discussion

In this study, candidate genes for CH previously identified through GWAS were validated by means of genetic analysis of missense variants, methylation and gene expression analysis. While *MERTK* has already been investigated more closely, we present here additional evidence for its implication in CH pathophysiology. For the first time, the use of direct biological tissue suggests functional changes in relation to CH for candidate genes *DUSP10*, *FTCDNL1*, *CAPN2*, *FHL5/UFL1* and *LRP1*. However, two candidate genes; *WNT2* and *PLCE1* could not be validated in our study.

While the consequences of changes in methylation are difficult to predict, altered gene expression is a common outcome [30]. The effect depends on several factors such as location of the methylation site in comparison to the gene. Hypermethylation of the promoter region usually corresponds to a lower gene expression, while hypermethylation in the protein coding region can have different effects, including increasing gene expression [31]. The methylation sites analysed here were intronic, and the difference detected between the CH and control group is small, therefore predictions on the consequence of this change on this specific position cannot be made. What is shown by this analysis is that the change in methylation predicted by post-GWAS analysis in a previous article was relevant; in the previous study, genetically driven methylation was predicted to lead to hypermethylation of CpG site cg05979619 [9]. Here, higher methylation of a CpG site two base pairs downstream from cg05979619 was found. Furthermore, the coding variants rs2230515 and rs2273621 in *MERTK* respectively *FHL5* are two variants predicted to alter protein function and were suggested to represent functional variants in previous post-GWAS analysis [7, 9]. Here both variants were confirmed to associate with CH, and further constitutes eQTLs. The risk associated allele of rs2230515 causes an increase of *MERTK* expression, while the risk allele of rs2273621 causes a decrease of *FHL5* expression. This is in line with previous data from our team, suggesting increased MERTK expression in tissue from patients with CH [10], and could indicate that a genetic predisposition for higher *MERTK* expression increases the risk of developing CH.

We further present evidence of altered gene expression of GWAS candidate genes. *FTCDNL1* had a higher gene expression in tissue from participants with CH compared to controls, while *CAPN2*, *DUSP10*, *UFL1*, and *LRP1* had lower gene expression. Little is known today about the *FTCDNL1* gene that may explain the relation to headache. In contrast to the other genes studied here, the *FTCDNL1* gene was specifically upregulated in fibroblasts. The gene is widely expressed in the body, with the highest levels detected in the liver and in skeletal muscles [32]. There is no reported *FTCDNL1* eQTL data for the lead variant. Pruning of the NHGRI-EBI GWAS catalogue listed *FTCDNL1* associations to smoking initiation, attention deficit hyperactivity disorder (ADHD) and bipolar disorder [33], in addition to reports on osteoporosis and schizophrenia [17, 18]. Genetic correlations to smoking and ADHD were reported in our previous CH GWAS, and *FTCDNL1* may constitute a biological link between CH and these traits [9]. Furthermore, CH and bipolar disorder have phenotypic similarities in having strong circadian rhythm components and also being treated with lithium [34].

The effect alleles of *CAPN2* (rs10916600) and *DUSP10* (rs17011182) identified by meta-GWAS represent eQTLs for the same genes, showing lower gene expression of *CAPN2* and *DUSP10* in relation to the disease associated allele, which supports our data. It is worth noting that *CAPN2* was originally identified as a CH locus in a Taiwanese cohort [6]. Although replicated in a trans-ancestry meta-analysis, the *CAPN2* locus was not observed in any of the European cohorts alone, and the association was considered to be driven by the East Asian cohort [7–9]. Our results showing decreased gene expression levels of *CAPN2* suggest that though the locus did not reach genome wide significance in the European cohorts, *CAPN2* may still have relevance for CH patients of European origin. The *FHL5* and *UFL1* genes are closely located to each other, while the lead variant for the locus is located in *FHL5*, both genes reside within the same LD block surrounding the lead variant. *FHL5* is highly expressed in muscle and vascular tissue but not in blood cells or fibroblasts and could not be analysed here because of tissue availability. The CH associated variant rs9486725 in the *FHL5* gene is an eQTL for *UFL1*, with reported changes in gene expression in the same direction (lower *UFL1*) as in our results. Consequently, there is a likelihood that the *UFL1* gene has biological relevance for the disorder and might represent the true association at this locus. The *LRP1* variant rs11172113 has previously been associated with both CH and migraine. The disease associated genetic variants display more complex relationships as the eQTLs reported for *LRP1* in the GTEx portal have different directions and do not directly support our results of lower gene expression in blood. In summary, changes in gene expression were discovered in biological tissue from individuals with CH of several genes located to CH GWAS loci; *DUSP10*, *FTCDNL1*,* CAPN2*, *UFL1* and *LRP1*, in addition to *MERTK* that was validated previously. The changes demonstrated in *DUSP10*, *CAPN2*, *UFL1* correspond to the predicted changes from genetic associations and available eQTL data. The directionality of the changes in gene expression are subject to variations in different tissues and should preferably be validated also in nervous tissue before drawing any conclusions on the biological implications. When interpreting these data the effect of eQTLs and gene expression further needs to be weighted by the biological role of the protein (pro/anti-inflammatory or both) as well as presence of other isoforms, regulators and interactions with other proteins.

The analysis of pairwise gene expression correlation performed on gene expression data showed several significant correlations, specifically between *DUSP10*, CAPN2, *UFL1* and *LRP1* in blood cells (Supplementary Fig. 4B). These extensive associations suggest potential functional relationships, or that these genes may be subject to shared regulations. Furthermore, these genes are expressed in the same types of cells as per the Protein Atlas [32]. *DUSP10*, *CAPN2* and *UFL1* are ubiquitously expressed whereas *LRP1* (and *MERTK*) have more specific expression patterns and can be found at high levels in mononuclear phagocytes (due to their important role in phagocytosis). These genes have regulatory roles in important pathways such as phosphoinositide 3-kinase (PI3K)/AKT serine-threonine protein kinase (Akt) or NF-κB which in turn have decisive functions in cell behaviour and are involved in a multitude of normal and pathological cellular conditions, including apoptosis and cancer. In the context of immune cells, the signalling pathways often translate into steering the immune cell into an appropriate phenotype. For example, DUSP10 acts as a negative regulator of Mitogen-Activated Protein Kinase 14 (p38) and c-Jun N-terminal kinase (JNK), two pro-inflammatory proteins, that tunes the inflammatory response through dephosphorylation [16]. Interestingly, inhibitory activity of DUSP10 on extracellular signal-regulated kinase (ERK or mitogen-activated protein kinase (MAPK)) has been shown in non-small cell lung cancer cells [35]. This same MAPK is part of the downstream pathway of MERTK which, after activation through its ligand Gas6 may result in phosphorylation and activation of ERK [36, 37]. Therefore, a decrease in *DUSP10*, as seen in this study could theoretically lead to less negative control of the MERTK/ERK pathway in CH, which is consistent with the previously demonstrated increase of *MERTK* expression in CH [10]. CAPN2 has a less defined pro or anti-inflammatory role [38]. This calcium-dependent protease reacts to changes in Ca^2+^ and is crucial to a variety of immune activities such as pyroptosis, a programmed lytic form of cell death, or M1 (pro-inflammatory state) polarization in macrophages [39, 40]. Recently, a study showed repressive activity of CAPN2 on calpain-1 catalytic subunit (CAPN1) transcription. When CAPN2 was deleted, there was activation of pro-inflammatory cytokine release mediated through CAPN1 activity (which was no longer repressed) [41]. Similar consequences may occur in relation to the present results as CAPN2 was found to be downregulated. An immune modulatory role is defined for UFL1; specifically, UFL1 selects proteins to be UFMylated and is central to the function of this ubiquitin-like system [42]. Furthermore, UFL1 has been reported to be involved in cytokine release in T-cells and was shown to prevent pro-inflammatory polarization in macrophages after LPS-stimulation [43, 44]. Lastly, LRP1 can activate both inflammatory and anti-inflammatory pathways depending on the context and has been extensively studied in a wide array of immune cells [45]. There have been significant findings of inflammatory markers in tissue from individuals with CH previously [46]. Recently by means of cytokine arrays in two separate CH cohorts from Nordic countries, several cytokines were found to differ between individuals with CH and controls in both serum and cerebrospinal fluid [47, 48]. The links to inflammatory pathways highlighted by the candidate genes described above further underline the importance and relevance of investigating inflammation in relation to CH pathophysiology. Here, fibroblasts were used to investigate gene expression as disease relevant tissue was not available. These peripheral cells do not directly translate into physiological events observed in headache pathophysiology and specifically neuroinflammation. The blood brain barrier seems not to be affected in migraine [49], and there is no evidence suggesting that it is compromised in cluster headache. Even so, there is cross-talk between peripheral and central nervous tissue that relates inflammatory signals. Importantly the TG, key to headache pathophysiology, is located outside of the blood brain barrier and may be exposed to more factors stemming from peripheral inflammation [50]. Future studies may want to investigate how the different transcription profiles of immune related genes discovered here would affect the inflammatory cells of different key regions of the central nervous system in relation to inflammatory events of the periphery and central nervous system occurring in cluster headache.

**Fig. 4:**
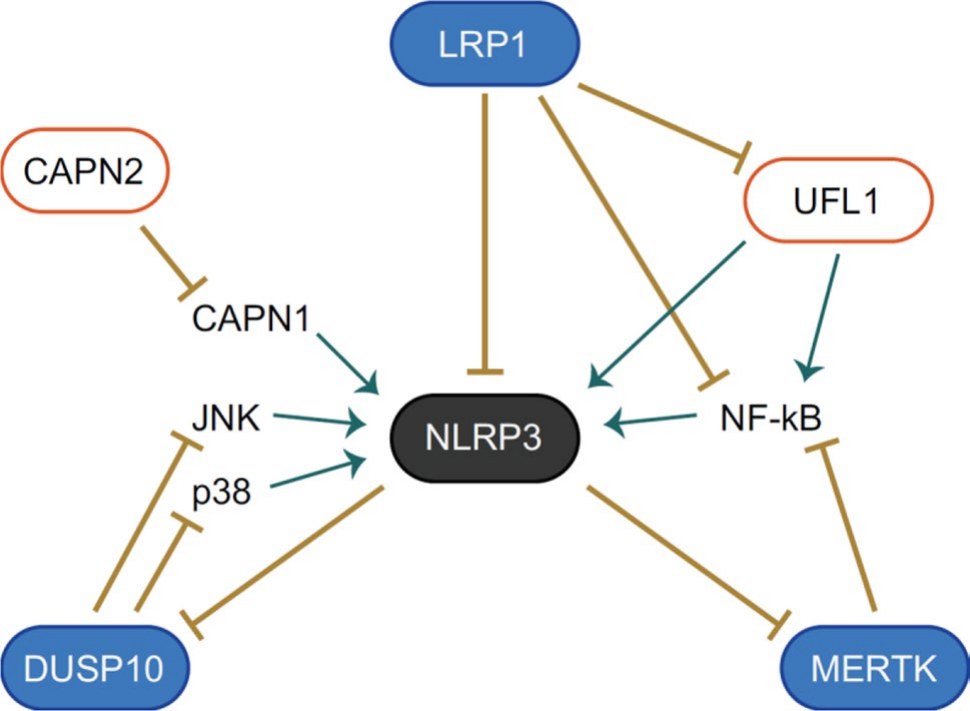
Hypothesized convergence of signaling on inflammasome. Pathway regulations of the different genes of interest on the activation of the NLRP3 inflammasome. The blue-coloured genes are anti-inflammatory, orange-framed genes have both pro- and anti-inflammatory functions. Brown blunt arrows show inhibition, green arrows activation. CAPN2/1; Calpain-1/2 catalytic subunit, DUSP10; dual specificity phosphatase 10, JNK; c-Jun N-terminal kinase, LRP1; low-density lipoprotein receptor-related protein, 1 MERTK; MER proto-oncogene tyrosine kinase, NF-κB; nuclear factor kappa B, NLRP3; NOD-, LRR- and pyrin domain- containing protein 3, p38; Mitogen-Activated Protein Kinase 14, UFL1; UFM1 Specific Ligase 1

While looking at the different pathways affected by the four genes correlating in blood; *DUSP10*, *CAPN2*, *UFL1* and *LRP1*, we observed their implication in the regulation of the activation of the NOD-, LRR- and pyrin domain- containing protein 3 (NLRP3) inflammasome (Fig. 4). The NLRP3 inflammasome is a protein complex that leads to the activation of its effective component: caspase-1. The downstream effect of caspase-1 promotes a pro-inflammatory environment through two parallel pathways; through the generation of the active form of IL-1β and IL-18, two pro-inflammatory cytokines, and additionally through the trigger of pyroptosis which induces the leak of cytoplasmic content into the extracellular space and thereby exacerbates the inflammatory response. The modulation of the NLRP3 inflammasome is critical and requires tailored modulation. So far, the non-canonical and direct activating pathways of NLRP3 have only been shown to happen through LPS-stimulation. The canonical pathway consists in a priming signal which is mainly regulated by NF-κB, a transcription factor and an activation signal that translocate to the nucleus when activated [51].

Both LRP1 and MERTK have been shown to inhibit the activation of NF-κB, and therefore also inhibit the activation of the NLRP3 inflammasome [52–54]. The strong anti-inflammatory role of LRP1 is supported through the repression of different pathways of NLRP3 activation [55, 56]. The relationship of UFL1 and NLRP3 is more complex as it seems that the cell environment dictates the types of modulation the UFMylation system triggers. For example, in LPS-stimulated mamillary epithelial cells, suppressing UFL1 exacerbates the inflammatory response through NLRP3 activation [57]. However, in another study carried out on macrophages, UFMylation of NLRP3 through UFL1/NLRP3 interaction, enhanced the priming signal of the inflammasome [58]. Similarly, calpains have been shown to have both positive and negative actions on NLRP3 activation. Mainly CAPN1 seems to activate NLRP3 while CAPN2 negatively regulates CAPN1 levels [59]. In the present study, where lower CAPN2 levels were observed, CAPN1 levels could potentially be higher, facilitating NLRP3 activation.

The different pathways discussed here have been demonstrated in different cell types and/or cell lines, which makes it difficult to predict how they would affect headache pathophysiology. More research is needed in order to determine how inflammatory signalling is involved in CH, and whether or not NLRP3 inflammasomes are implicated in these pathways. In support of the involvement of inflammasomes in CH, there is data showing altered IL-1β in CH (a first event of inflammasome activation); while IL-1β was low in active bouts in a Danish CH cohort, another study reported higher levels during attacks. While these data are circumstantial, inflammasomes have previously been discussed in relation to headache pathophysiology [60, 61]. Another interesting aspect is the implication of the molecular clock in NLRP3 regulation. Indeed, NLRP3, as an important regulator of the pro-inflammatory response, has been shown to be directly regulated by key molecular clock genes such as *BMAL1* and Nuclear Receptor Subfamily 1 Group D Member 1 (*NR1D1* or *Rev-erbα*) [62–65]. These interactions point to a promising path of future research as cluster headache is known for its circadian peculiarities [66, 67], and specifically the association of *BMAL1* along with several other molecular clock genes [68].

We have used robust methodology and tissue from individuals with CH, a majority of the study participants have a diagnosis validated by a neurologist. A small subset of study participants had self-reported CH (*n* = 62) which should also be considered when interpreting the results. Self-selection bias might occur, since patients with a higher disease burden may be more motivated to participate in a research study or, on the contrary, have less energy to participate. While GWAS, and genetic associations may detect correlations that are not directly linked to the pathology (they may for example reflect associations to a true disease-causing gene by LD), gene expression data as presented here, are likely to reflect a direct biological link to the pathophysiological events. A limitation of our study is small samples sizes, which affects statistical power and increases the chance of false negatives. *WNT2* should therefore not be excluded as potential disease-causing genes before being investigated in other larger independent materials. Small sample sizes may also overestimate effect sizes, introduce some degree of sampling error which may affect the generalizability of the results and studies with small sample sizes sometimes have poor reproducibility. However, study participants included in the gene expression analysis were all diagnosed by a neurologist and are well characterized prior to inclusion, which is a strength of our study. Another limitation is tissue availability. As biopsies of disease related tissue (nervous, vascular) were not an option in this study, skin and blood cells were used. Skin and blood cells were found to show different expression profiles which suggest that other relationships may be detected, for example, in nervous tissue. Additionally, *PLCE1* was not detected in the available tissues. Consequently, future studies may want to investigate these GWAS genes in independent cohorts, larger samples, and other tissues to further complete the picture of how these genes may affect the pathophysiology of CH. Also, protein and functional studies are warranted.

## Conclusions

The experimental data presented here validate *DUSP10*, *CAPN2*, *MERTK*, *FTCDNL1*, *FHL5/UFL1* and *LRP1* as CH candidate genes in peripheral tissue from individuals with CH. All these genes, except for *FTCDNL1*, can somehow be related to immune signalling pathways resulting in less pro-apoptotic signalling or less activation of inflammasomes, further strengthening the idea of inflammation as a driver in CH pathophysiology.

## Supplementary Information

Below is the link to the electronic supplementary material.

Supplementary Material 1

## Data Availability

The data used and analysed during the current study are available from the corresponding author on reasonable request.

## Abbreviations

ADHD: Attention deficit hyperactivity disorder
Akt: AKT serine-threonine protein kinase
CADD: Combined Annotation Dependent Depletion
CAPN1: Calpain-1 catalytic subunit
CAPN2: Calpain-2 catalytic subunit
CH: Cluster headache
CREB: cAMP response element-binding protein
DUSP10: Dual specificity phosphatase 10
ERK: Extracellular signal-regulated kinase
eQTL: Expression Quantitative Trait Loci
FDR: False discovery rate
FHL5: Four and a half LIM domains 5
FTCDNL1/FONG: Formiminotransferase cyclodeaminase N-terminal like
Gal: 3-Galectin-3
GWAS: Genome wide association study
HWE: Hardy-Weinberg equilibrium
I^2^: Heterogeneity
IPO8: Importin 8
JNK: c-Jun N-terminal kinase
LD: Linkage disequilibrium
LRP1: Low-density lipoprotein receptor-related protein 1
MAPK: Mitogen-activated protein kinase
MERTK: MER proto-oncogene tyrosine kinase
NF: κB-nuclear factor kappa B
NLRP3: NOD-, LRR-and pyrin domain-containing protein 3
NR1D1/Rev: erbα-Nuclear Receptor Subfamily 1 Group D Member 1
OR: Odds ratio
p38: Mitogen-Activated Protein Kinase 14
PI3K: Phosphoinositide 3-kinase
PIP2: Phosfatidylinositol-4,5-bisfosfate
PLCE1: Phospholipase C epsilon 1
𝜏^2^: Tau squared
TAM: TYRO3/AXL/MER
TBP: TATA-Box Binding Protein
TG: Trigeminal ganglion
UFL1: UFM1 Specific Ligase 1
WNT2: Wingless-type MMTV integration site family, member 2

## References

[1] Waung MW, Taylor A, Qualmann KJ, Burish MJ (2020) Family history of cluster headache: a systematic review. JAMA Neurol 77:887. 10.1001/jamaneurol.2020.0682 32310255 10.1001/jamaneurol.2020.0682 PMC7644512

[2] Fourier C, Ran C, Steinberg A et al (2022) Sex differences in clinical features, treatment, and lifestyle factors in patients with cluster headache. Neurology. 10.1212/WNL.0000000000201688 36543572 10.1212/WNL.0000000000201688 PMC10033163

[3] Leone M, Russell MB, Rigamonti A et al (2001) Increased familial risk of cluster headache. Neurology 56:1233–1236. 10.1212/WNL.56.9.1233 11342697 10.1212/wnl.56.9.1233

[4] Coppola G, Arruda MA, Ashina M et al (2025) Hallmarks of primary headache: part 3 – cluster headache. J Headache Pain 26:196. 10.1186/S10194-025-02145-6 41039211 10.1186/s10194-025-02145-6 PMC12490097

[5] Isayeva U, Paribello P, Ginelli E et al (2024) Genomics and pharmacogenomics of cluster headache: implications for personalized management? A systematic review. Psychiatr Genet 35:1. 10.1097/YPG.0000000000000380 39560176 10.1097/YPG.0000000000000380 PMC11698140

[6] Chen SP, Hsu CL, Wang YF et al (2022) Genome-wide analyses identify novel risk loci for cluster headache in Han Chinese residing in Taiwan. J Headache Pain 23. 10.1186/S10194-022-01517-6 10.1186/s10194-022-01517-6 PMC9677903 36404298

[7] O’Connor E, Fourier C, Ran C et al (2021) Genome-Wide association study identifies risk loci for cluster headache. Ann Neurol 90:193–202. 10.1002/ana.26150 34184781 10.1002/ana.26150 PMC7619354

[8] Harder AVE, Winsvold BS, Noordam R et al (2021) Genetic susceptibility loci in genomewide association study of cluster headache. Ann Neurol 90:203–216. 10.1002/ana.26146 34180076 10.1002/ana.26146 PMC8362054

[9] Winsvold BS, Harder AVE, Ran C et al (2023) Cluster headache genomewide association study and meta-analysis identifies eight loci and implicates smoking as causal risk factor. Ann Neurol 94:713–726. 10.1002/ana.26743 37486023 10.1002/ana.26743 PMC10952302

[10] Edvinsson JCA, Ran C, Olofsgård FJ et al (2024) MERTK in the rat trigeminal system: a potential novel target for cluster headache? J Headache Pain 25:85. 10.1186/s10194-024-01791-6 38783191 10.1186/s10194-024-01791-6 PMC11119394

[11] Fimia GM, Cesare D, De, Sassone-Corsi P (2000) A family of LIM-only transcriptional coactivators: tissue-specific expression and selective activation of CREB and CREM. Mol Cell Biol 20:8613. 10.1128/MCB.20.22.8613-8622.2000 11046156 10.1128/mcb.20.22.8613-8622.2000 PMC102166

[12] Mitsikostas DD, Knight YE, Lasalandra M et al (2011) Triptans attenuate capsaicin-induced CREB phosphorylation within the trigeminal nucleus caudalis: a mechanism to prevent central sensitization? J Headache Pain 12:412:411–417. 10.1007/S10194-011-0352-2 10.1007/s10194-011-0352-2 PMC3139063 21626018

[13] Wang J, Gao T, Zhang D et al (2025) Phospholipase C epsilon 1 as a therapeutic target in cardiovascular diseases. J Adv Res 77:235–256. 10.1016/j.jare.2025.01.032 39855298 10.1016/j.jare.2025.01.032 PMC12627389

[14] Dusaban SS, Purcell NH, Rockenstein E et al (2013) Phospholipase C epsilon links G protein-coupled receptor activation to inflammatory astrocytic responses. Proc Natl Acad Sci U S A 110:3609–3614. 10.1073/pnas.1217355110 23401561 10.1073/pnas.1217355110 PMC3587233

[15] Yancey PG, Blakemore J, Ding L et al (2010) Macrophage LRP-1 controls plaque cellularity by regulating efferocytosis and Akt activation. Arterioscler Thromb Vasc Biol 30:787–795. 10.1161/ATVBAHA.109.202051 20150557 10.1161/ATVBAHA.109.202051 PMC2845445

[16] Jiménez-Martínez M, Stamatakis K, Fresno M (2019) The dual-specificity phosphatase 10 (DUSP10): its role in cancer, inflammation, and immunity. Int J Mol Sci 20. 10.3390/IJMS20071626 10.3390/ijms20071626 PMC6480380 30939861

[17] Dókus LE, Yousef M, Bánóczi Z (2020) Modulators of calpain activity: inhibitors and activators as potential drugs. Expert Opin Drug Discov 15:471–486. 10.1080/17460441.2020.1722638 32022614 10.1080/17460441.2020.1722638

[18] Wang H, Gilner JB, Bautch VL et al (2007) Wnt2 coordinates the commitment of mesoderm to hematopoietic, endothelial, and cardiac lineages in embryoid bodies. J Biol Chem 282:782–791. 10.1074/JBC.M606610200 17098737 10.1074/jbc.M606610200

[19] Headache Classification Committee of the International Headache Society (IHS) (2013) The international classification of headache disorders, 3rd edition (beta version). Cephalalgia 33:629–808. 10.1177/0333102413485658 10.1177/0333102413485658 23771276

[20] Auton A, Abecasis GR, Altshuler DM et al (2015) A global reference for human genetic variation. Nat 2015 526:7571. 10.1038/nature15393 10.1038/nature15393 PMC4750478 26432245

[21] Fourier C, Ran C, Zinnegger M et al (2018) A genetic CLOCK variant associated with cluster headache causing increased mRNA levels. Cephalalgia 38:496–502. 10.1177/0333102417698709 28466652 10.1177/0333102417698709

[22] Ye J, Coulouris G, Zaretskaya I et al (2012) Primer-BLAST: a tool to design target-specific primers for polymerase chain reaction. BMC Bioinformatics 13:134. 10.1186/1471-2105-13-134 22708584 10.1186/1471-2105-13-134 PMC3412702

[23] Untergasser A, Cutcutache I, Koressaar T et al (2012) Primer3-new capabilities and interfaces. Nucleic Acids Res 40:e115. 10.1093/nar/gks596 22730293 10.1093/nar/gks596 PMC3424584

[24] Koressaar T, Remm M (2007) Enhancements and modifications of primer design program Primer3. 23:1289–1291. 10.1093/bioinformatics/btm091 10.1093/bioinformatics/btm091 17379693

[25] Purcell S, Neale B, Todd-Brown K et al (2007) PLINK: a tool set for whole-genome association and population-based linkage analyses. Am J Hum Genet 81:559–575. 10.1086/519795 17701901 10.1086/519795 PMC1950838

[26] Schloerke B, Cook D, Larmarange J et al (2025) GGally: extension to ggplot2

[27] Lonsdale J, Thomas J, Salvatore M et al (2013) The genotype-tissue expression (GTEx) project. Nat Genet 45:580. 10.1038/NG.2653 23715323 10.1038/ng.2653 PMC4010069

[28] Suzuki MM, Bird A (2008) DNA methylation landscapes: provocative insights from epigenomics. Nat Rev Genet 9:465–476. 10.1038/nrg2341 18463664 10.1038/nrg2341

[29] Jones PA (2012) Functions of DNA methylation: islands, start sites, gene bodies and beyond. Nature Reviews Genetics 13:713:484–492. 10.1038/nrg3230 10.1038/nrg3230 22641018

[30] Uhlén M, Fagerberg L, Hallström BM et al (2015) Tissue-based map of the human proteome. Science (1979) 347. 10.1126/science.1260419 10.1126/science.1260419 25613900

[31] Cerezo M, Sollis E, Ji Y et al (2025) The NHGRI-EBI GWAS Catalog: standards for reusability, sustainability and diversity. Nucleic Acids Res 53:D998–D1005. 10.1093/NAR/GKAE1070 39530240 10.1093/nar/gkae1070 PMC11701593

[32] Kou I, Takahashi A, Urano T et al (2011) Common variants in a novel gene, FONG on chromosome 2q33.1 confer risk of osteoporosis in Japanese. PLoS ONE 6. 10.1371/JOURNAL.PONE.0019641 10.1371/journal.pone.0019641 PMC3089633 21573128

[33] Li S, Li J, Liu J et al (2021) Regulatory variants at 2q33.1 confer schizophrenia risk by modulating distal gene TYW5 expression. Brain 145:770. 10.1093/brain/AWAB357 10.1093/brain/awab357 PMC9014752 34581804

[34] Robbins MS (2013) The psychiatric comorbidities of cluster headache. Curr Pain Headache Rep 17:313. 10.1007/S11916-012-0313-8/23296640 10.1007/s11916-012-0313-8

[35] Wei X, Png CW, Weerasooriya M et al (2023) Tumor promoting function of DUSP10 in non-small cell lung cancer is associated with tumor-promoting cytokines. Immune Netw 23. 10.4110/IN.2023.23.E34 10.4110/in.2023.23.e34 PMC10475826 37670811

[36] Miao L, Yu C, Guan G et al (2024) Extracellular vesicles containing GAS6 protect the liver from ischemia-reperfusion injury by enhancing macrophage efferocytosis via MerTK-ERK-COX2 signaling. Cell Death Discov 10:401. 10.1038/S41420-024-02169-Y 39256347 10.1038/s41420-024-02169-y PMC11387478

[37] Cai B, Kasikara C, Doran AC et al (2018) MerTK signaling in macrophages promotes the synthesis of inflammation resolution mediators by suppressing CaMKII activity. Sci Signal 11. 10.1126/SCISIGNAL.AAR3721 10.1126/scisignal.aar3721 PMC6171110 30254055

[38] Kumar V, Stewart JH (2025) Viewing inflammation and immunoregulation under the calpain system lens. Cells 14:1814 14:1814. 10.3390/CELLS14221814 10.3390/cells14221814 PMC12650998 41294867

[39] Zhang L, Zheng D, Yan Y et al (2022) Myeloid cell-specific deletion of Capns1 prevents macrophage polarization toward the M1 phenotype and reduces interstitial lung disease in the bleomycin model of systemic sclerosis. Arthritis Res Ther 24. 10.1186/S13075-022-02833-7 10.1186/s13075-022-02833-7 PMC9210712 35729674

[40] Davis MA, Fairgrieve MR, Den Hartigh A et al (2019) Calpain drives pyroptotic vimentin cleavage, intermediate filament loss, and cell rupture that mediates immunostimulation. Proc Natl Acad Sci U S A 116:5061–5070. 10.1073/PNAS.1818598116 30796192 10.1073/pnas.1818598116 PMC6421439

[41] Binte Hanafi Z, Mei Y, Teo HY et al (2025) Calpain 2 regulates IL-1α secretion and inhibits tumor development via modulating calpain 1 expression in the tumor microenvironment. Oncoimmunology 14. 10.1080/2162402X.2025.2451444 10.1080/2162402X.2025.2451444 PMC11730618 39803956

[42] Liang Z, Ning R, Wang Z et al (2024) The emerging roles of UFMylation in the modulation of immune responses. Clin Transl Med 14. 10.1002/CTM2.70019 10.1002/ctm2.70019 PMC11389534 39259506

[43] Wang S, Liu Y, Su M et al (2024) UFMylation is involved in serum inflammatory cytokines generation and splenic T cell activation induced by lipopolysaccharide. Cytokine 183:156755. 10.1016/J.CYTO.2024.156755 39276536 10.1016/j.cyto.2024.156755

[44] Balce DR, Wang YT, McAllaster MR et al (2021) UFMylation inhibits the proinflammatory capacity of interferon-γ–activated macrophages. Proc Natl Acad Sci U S A 118. 10.1073/PNAS.2011763118 10.1073/pnas.2011763118 PMC7817147 33372156

[45] Sizova O, John LS, Ma Q, Molldrem JJ (2023) Multi-faceted role of LRP1 in the immune system. Front Immunol 14. 10.3389/FIMMU.2023.1166189 10.3389/fimmu.2023.1166189 PMC10069629 37020553

[46] Søborg MLK, Jensen RH, Barloese M, Petersen AS (2024) Biomarkers in cluster headache: A systematic review. Headache: J Head Face Pain 64:98–116. 10.1111/HEAD.14641 10.1111/head.14641 38111226

[47] Lund NLT, Westgate CSJ, Søborg MLK et al (2025) Distinct Alterations of Inflammatory Biomarkers in Cluster Headache: A Case Control Study. Ann Neurol 98:4–18. 10.1002/ANA.27205 39981939 10.1002/ana.27205 PMC12174737

[48] Ran C, Olofsgård FJ, Wellfelt K et al (2024) Elevated cytokine levels in the central nervous system of cluster headache patients in bout and in remission. J Headache Pain 25. 10.1186/S10194-024-01829-9 10.1186/s10194-024-01829-9 PMC11267889 39044165

[49] Yamanaka G, Suzuki S, Morishita N et al (2021) Role of Neuroinflammation and Blood-Brain Barrier Permutability on Migraine. Int J Mol Sci 22:8929. 10.3390/IJMS22168929 34445635 10.3390/ijms22168929 PMC8396312

[50] Eftekhari S, Salvatore CA, Johansson S et al (2015) Localization of CGRP, CGRP receptor, PACAP and glutamate in trigeminal ganglion. Relation to the blood–brain barrier. Brain Res 1600:93–109. 10.1016/J.BRAINRES.2014.11.031 25463029 10.1016/j.brainres.2014.11.031

[51] Swanson KV, Deng M, Ting JPY (2019) The NLRP3 inflammasome: molecular activation and regulation to therapeutics. Nat Rev Immunol 19:477. 10.1038/S41577-019-0165-0 31036962 10.1038/s41577-019-0165-0 PMC7807242

[52] Su X, Chen W, Fu Y et al (2024) Protective Role of MerTK in Diabetic Peripheral Neuropathy via Inhibition of the NF-κB Signaling Pathway. Exp Clin Endocrinol Diabetes 132:396. 10.1055/A-2301-3970 38588709 10.1055/a-2301-3970 PMC11251753

[53] Yang L, Liu CC, Zheng H et al (2016) LRP1 modulates the microglial immune response via regulation of JNK and NF-κB signaling pathways. J Neuroinflammation 2016 13(1):304. 10.1186/S12974-016-0772-7 10.1186/s12974-016-0772-7 PMC5146875 27931217

[54] Guo X, Yang F, Liu T et al (2024) Loss of LRP1 Promotes Hepatocellular Carcinoma Progression via UFL1-Mediated Activation of NF-κB Signaling. Adv Sci 11:2401672. 10.1002/ADVS.202401672 10.1002/advs.202401672 PMC11615765 39405202

[55] Qiu WQ, Pan R, Tang Y et al (2020) Lychee seed polyphenol inhibits Aβ-induced activation of NLRP3 inflammasome via the LRP1/AMPK mediated autophagy induction. Biomed Pharmacother 130. 10.1016/J.BIOPHA.2020.110575 10.1016/j.biopha.2020.110575 32768883

[56] Yang CJ, Li X, Feng XQ et al (2022) Activation of LRP1 ameliorates cerebral ischemia/reperfusion injury and cognitive decline by suppressing neuroinflammation and oxidative stress through TXNIP/NLRP3 signaling pathway in mice. Oxid Med Cell Longev. 10.1155/2022/8729398 10.1155/2022/8729398 PMC9410841 36035210

[57] Li C, Wang X, Kuang M et al (2019) UFL1 modulates NLRP3 inflammasome activation and protects against pyroptosis in LPS-stimulated bovine mammary epithelial cells. Mol Immunol 112:1–9. 10.1016/j.molimm.2019.04.023 31078114 10.1016/j.molimm.2019.04.023

[58] Jing J, Yang F, Wang K et al (2025) UFMylation of NLRP3 Prevents Its Autophagic Degradation and Facilitates Inflammasome Activation. Adv Sci 12:2406786. 10.1002/advs.202406786 10.1002/advs.202406786 PMC12005806 39985286

[59] Liu X, Li M, Chen Z et al (2022) Mitochondrial calpain-1 activates NLRP3 inflammasome by cleaving ATP5A1 and inducing mitochondrial ROS in CVB3-induced myocarditis. Basic Res Cardiol 117. 10.1007/S00395-022-00948-1 10.1007/s00395-022-00948-1 PMC9399059 35997820

[60] Kursun O, Yemisci M, van den Maagdenberg AMJM, Karatas H (2021) Migraine and neuroinflammation: the inflammasome perspective. J Headache Pain 22:55. 10.1186/S10194-021-01271-1 34112082 10.1186/s10194-021-01271-1 PMC8192049

[61] Şahin E, Karaaslan Z, Şanlı E et al (2022) Reduced expression of inflammasome complex components in cluster headache. Headache 62:967–976. 10.1111/HEAD.14334 35670197 10.1111/head.14334

[62] Kou L, Chi X, Sun Y et al (2022) The circadian clock protein Rev-erbα provides neuroprotection and attenuates neuroinflammation against Parkinson’s disease via the microglial NLRP3 inflammasome. J Neuroinflammation 19. 10.1186/S12974-022-02494-Y 10.1186/s12974-022-02494-y PMC9169406 35668454

[63] Ren Y, Lv D, Chen J et al (2026) BMAL1 downregulation exacerbates age-related nonalcoholic steatohepatitis by promoting NLRP3 inflammasome activation via HIF-1ɑ-mediated glycolysis. Free Radic Biol Med 246:562–579. 10.1016/j.freeradbiomed.2026.01.058 41621599 10.1016/j.freeradbiomed.2026.01.058

[64] Li F, Lin L, He Y et al (2022) BMAL1 regulates *Propionibacterium acnes*-induced skin inflammation via REV-ERBα in mice. Int J Biol Sci 18:2597–2608. 10.7150/IJBS.71719 35414779 10.7150/ijbs.71719 PMC8990455

[65] Pourcet B, Zecchin M, Ferri L et al (2018) Nuclear Receptor Subfamily 1 Group D Member 1 Regulates Circadian Activity of NLRP3 Inflammasome to Reduce the Severity of Fulminant Hepatitis in Mice. Gastroenterology 154:1449–1464e20. 10.1053/j.gastro.2017.12.019 29277561 10.1053/j.gastro.2017.12.019 PMC5892845

[66] Ran C, Spulber S, Belin AC (2026) Chronobiology and cluster headache: insights into a hypothalamic disorder. Curr Opin Neurol 39. 10.1097/WCO.0000000000001466 10.1097/WCO.0000000000001466 41709685

[67] Burish M, Kim SA, Ran C et al (2025) Reviewing the complex relationship between circadian rhythms and cluster headache. Cephalalgia 45. 10.1177/03331024251365858 10.1177/03331024251365858 PMC12960194 40808422

[68] Sanches CD, Spulber S, Olofsgård FJ et al (2026) Genetic variability within molecular core clock genes in cluster headache. Cephalalgia 46. 10.1177/03331024261440136 10.1177/03331024261440136 41967867

