# Supplementary material for: Genome-wide association susceptibility loci for cluster headache support a role for inflammation in the pathophysiology
Source: J Headache Pain. 2026 Aug 4;27(1):198. doi: 10.1186/s10194-026-02485-x (PMC13440057; doi:10.1186/s10194-026-02485-x)
Supplement: Supplementary file 1 [file 10194_2026_2485_MOESM1_ESM.pdf]

Validation of genome-wide association susceptibility loci for cluster headache support a role for inflammation in the pathophysiology

Supplementary information

Table of Contents

Supplementary Table 1: Primers for gene expression, Pyro-sequencing and genotyping 2

Supplementary Table 2: Analysis of rs2273621, rs2274224 and rs2230515 in GWAS cohort.................... 3

Supplementary Table 3: Allele frequencies of rs2273621, rs2274224 and rs2230515 in subgroups of the replication cohort.. 3

Supplementary Figure 1: Study design 4

Supplementary Figure 2: Methylation analysis by Pyrosequencing 5

Supplementary Figure 3: eQTL plots for rs2230515 and rs2273621 6

Supplementary Figure 4: Correlation analysis of relative gene expression 7

Supplementary Table 1: Primers for gene expression, Pyro-sequencing and genotyping

Genes of interest	Chr	Forward primer 5'-3'	Reverse primer 3'-5'
DUSP10	1	CGAGTGAATGGGGGCTGAAT	CAGGAGGGTGGCTGTTACTG
FTCDNL1	2	GTCTTCTTCCAGAGTGGGGC	CAGCACCAGATCCTCAGCAA
CAPN2	2	GAGTCGCCCCGACCTTTCTC	GCCTCGTAGTCCTGGTTGAG
FHL5	6	CATTCTGCGTGGACTGCTAC	CGCAACTGAGTTCTGGCTTT
UFL1	6	AGCACGTATCCGTGGACTAT	GAAGCTGCTCCTGAAATCCA
WNT2	7	CTCCCTCTGCTCTTGACCTG	TTATCGCACATCACCCTGGA
PLCE1	10	GAGCTGCAATCGAAGTCTGG	AAGGCCTTCTGTGAGTCCTC
LRP1	12	GCATCCTGATTGAGCACCTG	GTTCACACGGATCACACTCG
**House-keeping genes**		**Forward primer 5'-3'**	**Reverse primer 3'-5'**
TBP	6	AGGCAACACAGGGAACCTC	TTGCAGCTGCGGTACAATCC
IPO8	12	ATTGGAAGAAACCGCGCTTG	TGTGTACACCTCCTGCAGTG
**Pyro-sequencing primers**		**Forward primer 5'-3'**	**Reverse primer 3'-5'**
MERTK	2	GTAGAAATGGGGTTTTATTATG TTGGTTAG<br>**Sequencing primer 5'-3'**<br>AGTGTTGGGATTATAGG	CAAAAATTAACCTCTATACTCCT TCTTATC
**TaqMan**		**SNP**	**Assay ID**
MERTK	2	rs2230515	C_27487306_10
FHL5	5	rs2273621	C_2810742_10
PLCE1	10	rs2274224	C_3137872_20

Chr = Chromosome, DUSP-10 = Dual specificity phosphatase 10, MERTK = MER proto-oncogene tyrosine kinase, FTCDNL1 = Formiminotransferase cyclodeaminase N-terminal like, FHL5 = Four and a half LIM domains 5, UFL1 = UFM1 specific ligase 1, WNT2 = Wingless-type MMTV integration site family, member 2, PLCE1 = Phospholipase C epsilon 1, LRP1 = Low-density lipoprotein receptor-related protein 1, CAPN2 = Calpain-2 catalytic subunit, TBP = TATA-binding protein, IPO8 = Importin 8

Supplementary Table 2: Analysis of rs2273621, rs2274224 and rs2230515 in GWAS cohort

Gene	Variant	Allele	Frequency % (n)		*OR* (*95% CI*)	Adj *P*
			CH patients	Controls		
**MERTK**	rs2230515	G	71.42 (767)	60 (348)	0.6 (0.48–0.76)	0.000077
		A	28.58 (307)	40 (232)		
**FHL5**	rs2273621	A	61.71 (643)	66.84 (393)	1.28 (1.01–1.63)	0.12
		G	38.29 (399)	33.16 (195)		
**PLCE1**	rs2274224	G	65.13 (693)	61.19 (361)	0.92 (0.73–1.16)	< 1
		C	34.87 (371)	38.81 (229)		

All three variants were in HWE: rs2230515 P = 0.69, rs2273621 P = 0.6 and rs2274224 P < 1. Logistic regression (additive model) with sex as covariate was utilised to analyse allele association. CH = cluster headache, OR = Odds ratio, CI = Confidence interval. Adj P = P-value adjusted for multiple testing using Bonferroni correction.

Supplementary Table 3: Allele frequencies of rs2273621, rs2274224 and rs2230515 in subgroups of the replication cohort

Gene	Variant	Allele	Allele frequency % (n)			
			CH all	CH confirmed	CH not confirmed	Controls
**MERTK**	rs2230515	G	67.79 (221)	69.31 (140)	65.32 (81)	62.87 (635)
		A	32.21 (105)	30.69 (62)	34.68 (43)	37.13 (375)
**FHL5**	rs2273621	A	59.26 (192)	58.41 (118)	60.66 (74)	66.36 (657)
		G	40.74 (132)	41.58 (84)	39.34 (48)	33.64 (333)
**PLCE1**	rs2274224	G	59.81 (195)	64.85 (131)	51.61 (64)	59.34 (591)
		C	40.18 (131)	35.15 (71)	48.39 (60)	40.66 (405)

CH = cluster headache, confirmed = diagnosis confirmed by a neurologist, not confirmed = diagnosis validated by questionnaire data.

Supplementary Figure 1: Study design

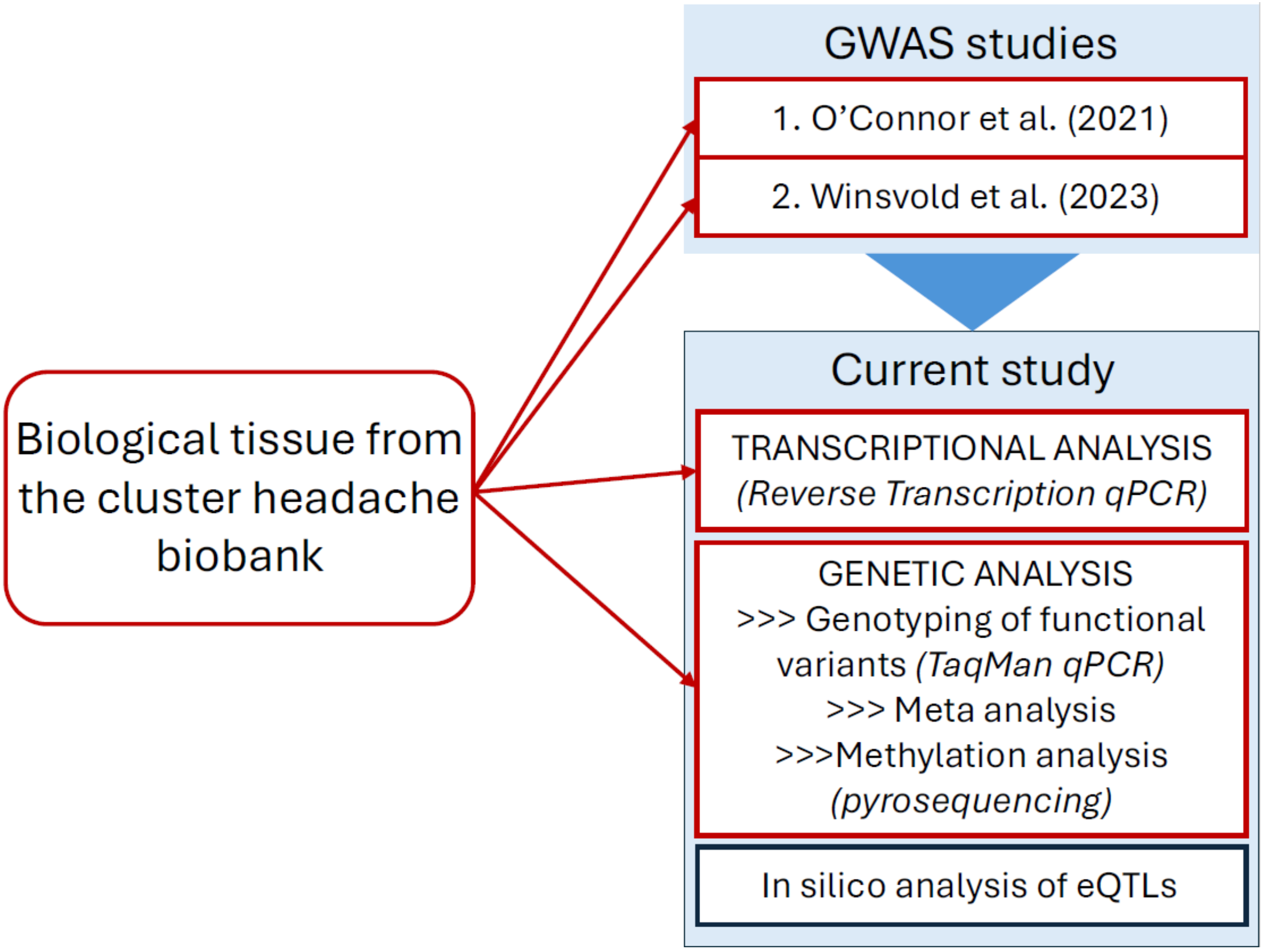

Supplementary Figure 2: Methylation analysis by Pyrosequencing

A: PCR amplification

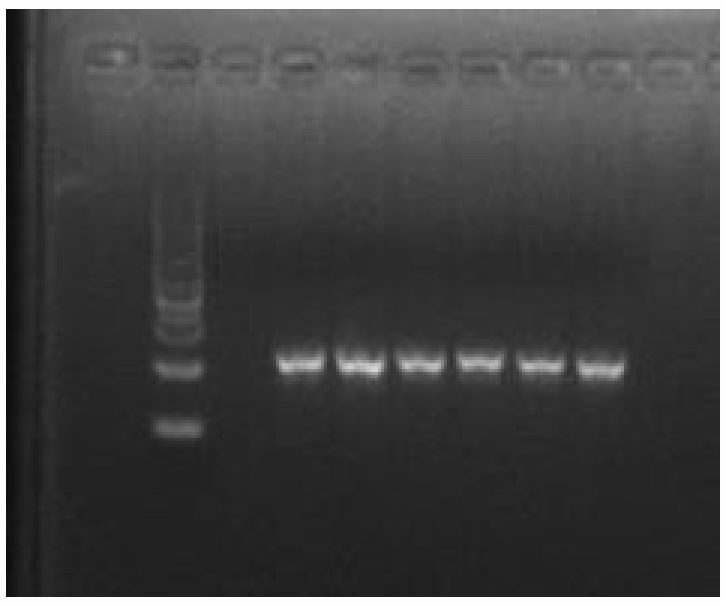

B: Pyro-methylation assay

Histogram

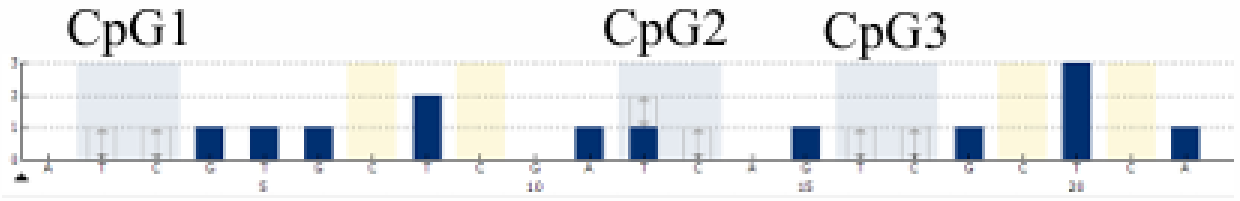

PYROgram

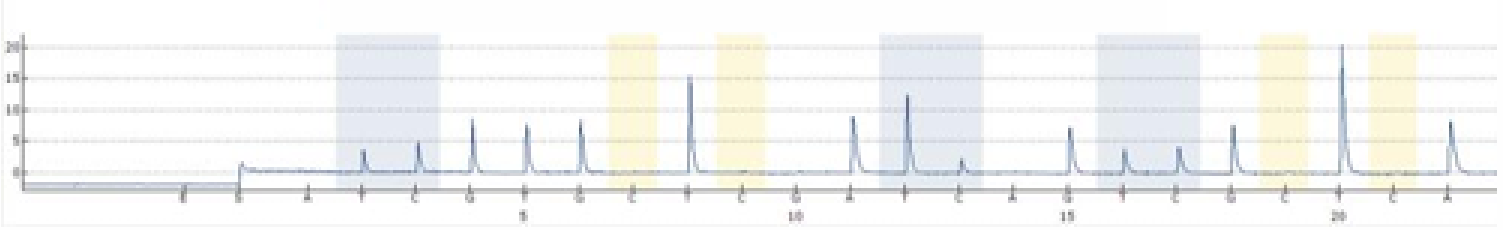

C: Methylation rate at each site

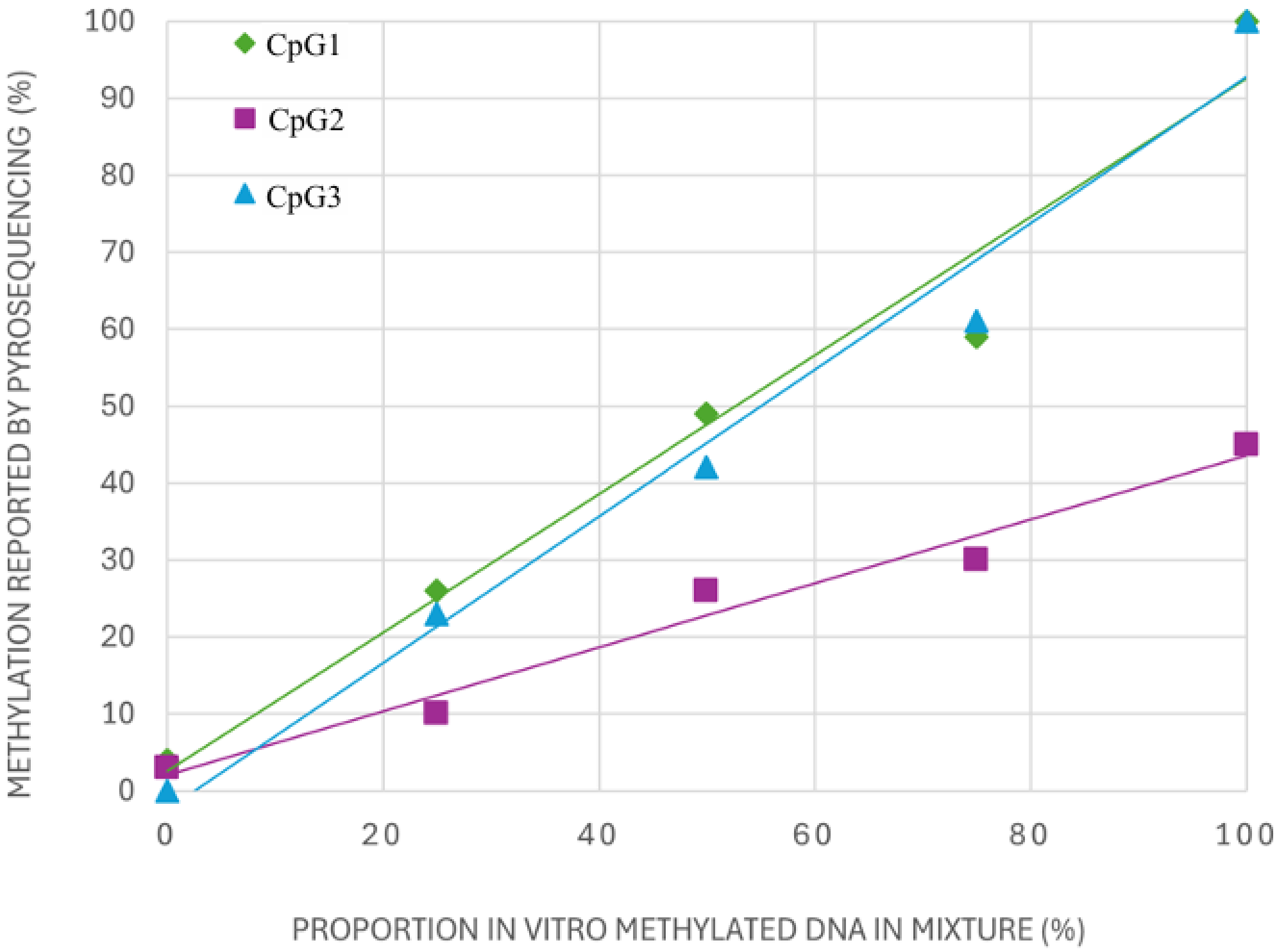

A: PCR product (219 bp) used to analyse methylation by Pyrosequencing. B: Methylation assay designed to capture the cg05979619 CpG site in the MERTK gene, position 112704577 on chromosome 2 (GRCh37 coordinates). The cg05979619 corresponds to Position 2 (CpG2) in the top panel (Histogram) and the bottom panel (example Pyrogram). C: Methylation assay test verifying a linear correlation for all three positions in test samples of methylated to unmethylated DNA at different ratios (0, 25, 50, 75 and 100 %).

Supplementary Figure 3: eQTL plots for rs2230515 and rs2273621

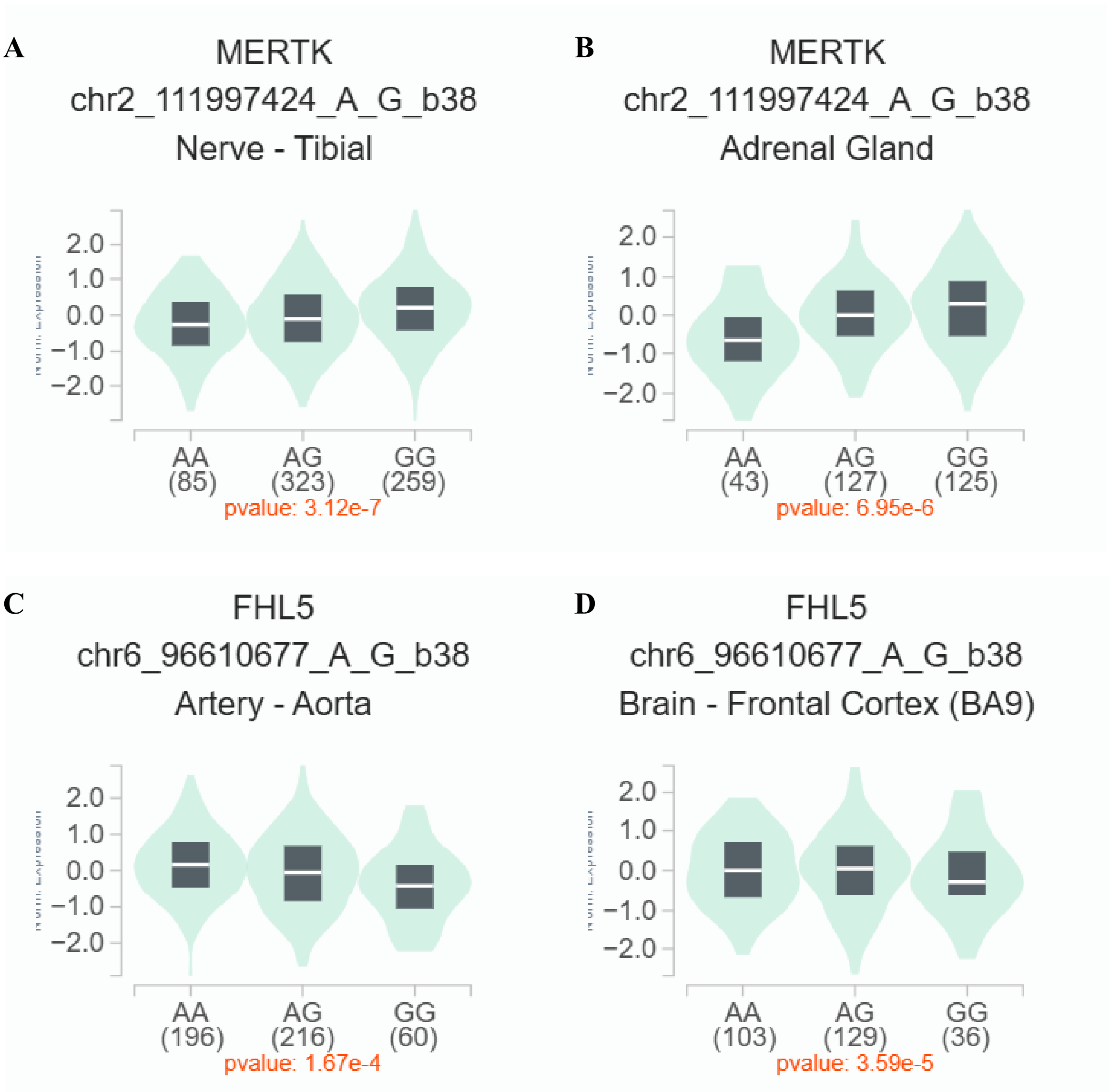

eQTL plots showing A-B: gene expression of MERTK (rs2230515) and C-D: FHL5 (rs2273621) showing that the risk allele in rs2230515 causes an increase of MERTK expression, while the risk allele of rs2273621 causes a decrease of FHL5 expression in different tissues. The Genotype-Tissue Expression (GTEx) Project was supported by the Common Fund of the Office of the Director of the National Institutes of Health, and by NCI, NHGRI, NHLBI, NIDA, NIMH, and NINDS.[1] The data for this report was obtained from GTEx Analysis Release V10 (dbGaP Accession phs000424.v10.p2) on 06/04/2026.

1. Lonsdale J, Thomas J, Salvatore M, et al. The Genotype-Tissue Expression (GTEx) project. *Nat Genet*. 2013;45(6):580. doi:10.1038/NG.2653

Supplementary Figure 4: Correlation analysis of relative gene expression

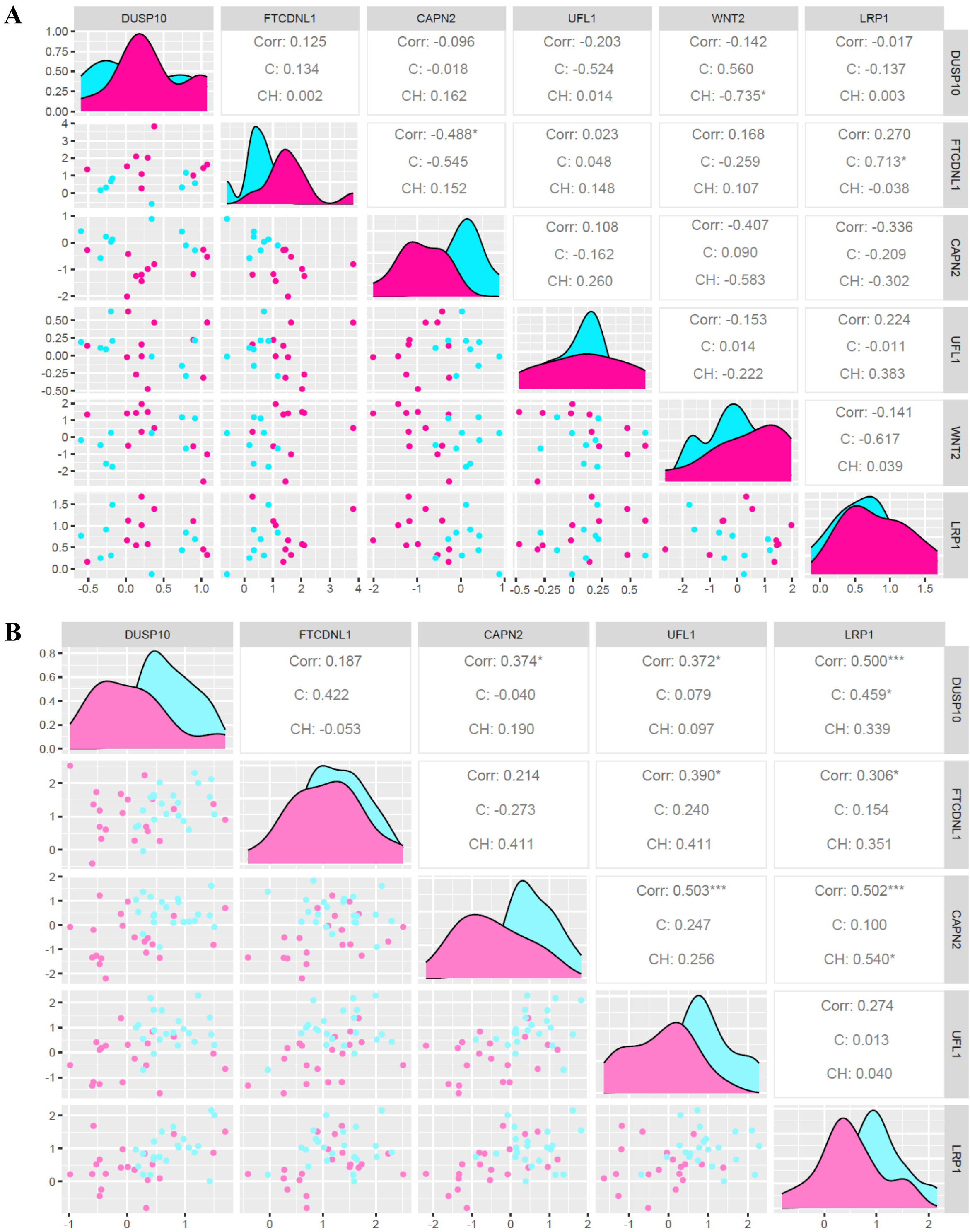

Correlation analysis by pairwise comparisons between relative gene expression for each candidate gene in fibroblasts (A) and in blood (B), controls (C) in blue, cluster headache (CH) in pink. DUSP-10 = Dual specificity phosphatase 10, FTCDNL1 = Formiminotransferase cyclodeaminase N-terminal like, CAPN2 = Calpain-2 catalytic subunit, UFL1 = UFM1 specific ligase 1, WNT2 = Wingless-type MMTV integration site family, member 2, LRP1 = Low-density lipoprotein receptor-related protein 1.
